# Development of an optimized cell-based selection system for phage display libraries

**DOI:** 10.1093/biomethods/bpaf009

**Published:** 2025-02-07

**Authors:** Malgorzata Czarnecka, Nicole Findik, Anja Schlör, Katja Hanack

**Affiliations:** Institute of Biochemistry and Biology, University of Potsdam, Karl-Liebknecht-Str. 24-25, D-14476 Potsdam, Germany; new/era/mabs GmbH, August-Bebel-Str. 89, 14482 Potsdam, Germany; new/era/mabs GmbH, August-Bebel-Str. 89, 14482 Potsdam, Germany; Institute of Biochemistry and Biology, University of Potsdam, Karl-Liebknecht-Str. 24-25, D-14476 Potsdam, Germany; new/era/mabs GmbH, August-Bebel-Str. 89, 14482 Potsdam, Germany

**Keywords:** antibody discovery, phage display, recombinant human antibodies, membrane proteins, selection

## Abstract

The discovery of antibodies through phage display is significantly influenced by antigen presentation during panning, particularly for membrane-anchored proteins, which pose challenges due to their complex structures. Traditional approaches, such as whole cells expressing the target protein, often result in low antigen density and high background signals. In this study, we describe an alternative method using stably transfected cell lines that express the target antigen on their surface, regulated by an intracellular enhanced green fluorescent protein (EGFP) signal. This system enables high-throughput flow cytometry-based screening of phage display libraries to isolate human antibodies that recognize the native conformation of membrane proteins. Using human epithelial cell adhesion molecule (EpCAM) and human neuroplastin 65 (NP65) as model antigens, we established an optimized screening workflow with polyclonal phage pools. Selected EpCAM-specific single-chain variable fragments (scFvs) from a naïve library were recombinantly expressed with an IgG4 scaffold and characterized for specific binding. This approach provides an effective platform for the identification of antibodies against membrane proteins in their native state.

## Introduction

Membrane proteins represent an interesting and important group of antigens which could be targeted with specific antibodies. During pathologic processes such as tumor cell growth they are over expressed and are therefore a suitable target or biomarker for antibody-based therapeutic or diagnostic applications. However, the generation of membrane protein specific antibodies is associated with major challenges because often these proteins are not available in their native conformation to be useful for antibody selection. As an alternative, recombinant expressed extracellular domains, linear peptide sequences, or over expressing cell lines were used for immunization approaches [1–4]. Inducing antigen-specific antibody responses *in vivo* is strongly dependent on how the antigen is presented. In addition, also for the selection process afterward, it is important that the test environment is mimicking the original environment as best as possible to find suitable binders for the final application. Recombinant expression of proteins or only parts of the protein alters the conformation and correct folding which can negatively impact the immunization result and later the screening process. Using linear peptide sequences coupled to carrier proteins is a common method used as alternative when the recombinant protein is not available or highly expensive. The problem with this approach is the linearity of the sequence. Finding antibodies that recognize the linear peptide sequence is easy, but in the original environment where the membrane protein is expressed on cells in a specific conformation, this sequence may be covered or hidden, preventing the antibody from finding its epitope. Another aspect to keep in mind is that for an ELISA-based screening of possible antibody candidates, the target proteins or peptides have to be coated on a solid phase. This process can lead to changes in the structure or to an overlapping, resulting in the coverage of epitope sequences and negative results during screening [5–7]. Especially in phage display workflows, this can lead to a lack of enrichment of specific phages. To circumvent these problems, over-expressing cell lines or targeted cancer cell lines were used for selection approaches to ensure a correct folding and presentation of the desired target on the cell surface [8, 9]. However, immunizing whole cells is reducing the specificity of the immune response to the desired target because the cell surface itself provides so many different epitopes that a broad polyclonal response is induced rather than a specific one for the target of interest.

As published previously, Jones *et al*. showed a cell-based phage display approach using mammalian cells transiently over expressing the target of interest fused to GFP [10]. In this study, human scFv antibodies reformatted to whole human IgG1 were selected against three membrane proteins, human CD83, canine CD117, and bat CD11b using the cell-based phage display protocol. The human naïve scFv library was incubated with the cell mix of transfected and non-transfected mammalian cells. Moreover, the antigen-specific bound phage particles were eluted according to the high GFP expression and its flow cytometric signal.

We decided to perform several adaptations to establish a modified approach and, therefore, improve the workflow of a cell-based phage display. In our study, the antigen-specific phage binding to the target expressed on the cell surface was detected with an anti-phage antibody conjugated to a fluorescent dye. Therefore, we determined the minimal amount of anti-phage antibody using the monoclonal phage library representing murine anti-fluoresceinisothiocyanate (FITC) B13DE1-scFv fragments and a stable transgenic human embryonic kidney 293 (HEK293) cell line labeled with streptavidin-FITC (SAV-FITC) as model antigen [11, 12]. Second, the minimal amount of phage particles was determined to detect the specific binding. For this purpose, we generated a polyclonal phage library with a known antibody sequence specific to FITC mimicking the selection round of a tangible phage display process.

**Figure 1:**
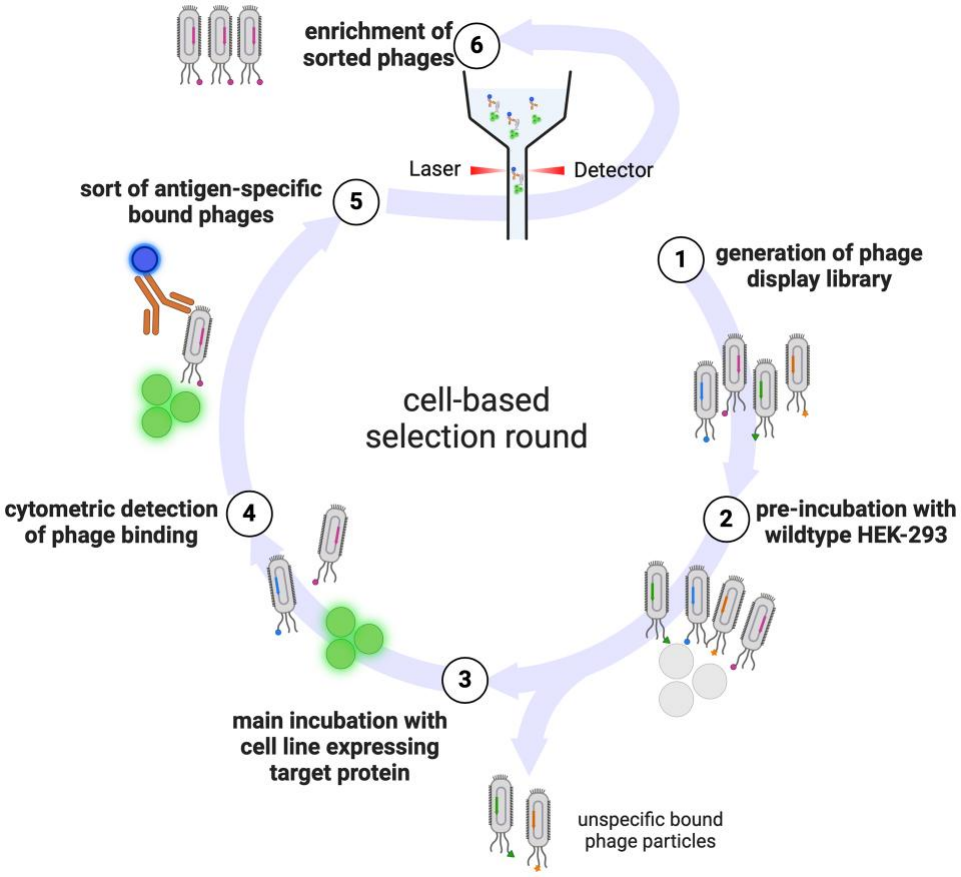
Schematic overview of the cell-based selection round. In the first step the phage display library is generated. Next, the phage library is pre-incubated with wild type HEK293 cells to eliminate the phage particles that bind unspecific to the cell surface. The unbound phage particles are incubated with the cell line expressing target membrane protein. The antigen-specific phage binding is detected with an anti-phage antibody conjugated to a fluorescent dye in a flow cytometer. In the last step phages are sorted and enriched. Created in https://BioRender.com/k48e008

Taking the insights of this model approach, an optimized cell-based and flow cytometric phage display workflow was established to identify and select phages from a naïve human phage display library specific for the human membrane proteins EpCAM and NP65. EpCAM is a type I transmembrane glycoprotein on healthy epithelial cells but over expressed during tumor growth [13]. NP65 is a type I transmembrane glycoprotein as well which belongs to the immunoglobulin superfamily and is expressed in the central nervous system and the cardiac conduction system [14, 15]. Using these antigens, an optimized flow cytometric selection workflow was used to select antigen-specific phages from a library in a high-throughput and time saving manner (Fig. 1).

## Materials and methods

### Generation of EpCAM and neuroplastin 65 overexpressing cell lines

DNA sequences from EpCAM (NM_002354.3) and NP65 (AF109126.1) were taken from the *National Library of Medicine.* For NP65, the serin at position two was substituted with an alanine. The whole cytoplasmic domains (aa 289-314 for intracellular domain of EpCAM and aa 360-393 for intracellular domain of neuroplastin-65) were exchanged with EGFP to have a transfection and sorting control. Using the *in silico* tool DTY Health Tech, TMHMM-2.0, the location of the transmembrane helices were predicted [16].

**Figure 2:**
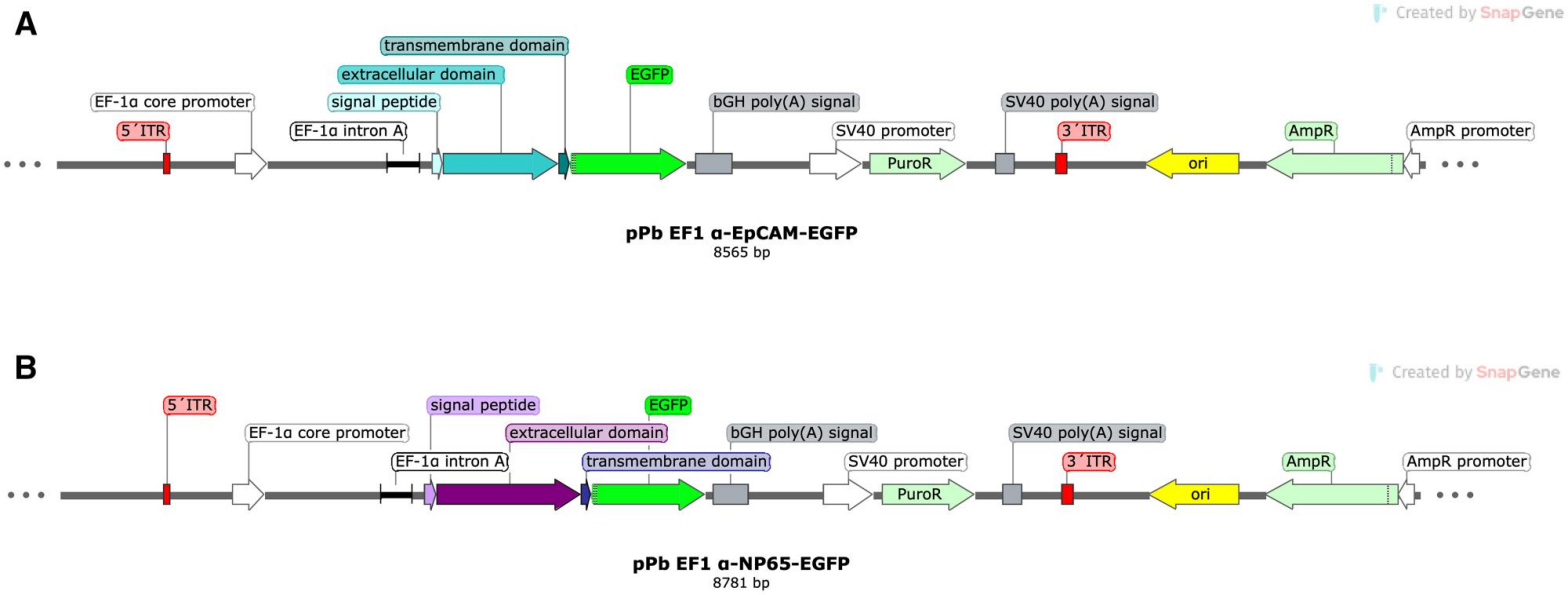
Vector maps of pPb EF1α-EpCAM-EGFP (**A**) and pPb EF1α-NP65-EGFP (**B**). The expression of both fusion proteins EpCAM-EGFP and NP65-EGFP was under control of EF1α promoter. Both expression vectors encoded for puromycin resistance gene. DNA sequences which should be integrated into the host genome were flanked by inverted terminal repeat (ITR, red) sequences. They were recognized by piggybac transposase (encoded on a separate helper plasmid), cutted out and integrated into TTAA chromosomal sites of host genome. For vector propagation in *E.coli* strains, both expression vectors possessed bacterial origin of replication (ori, yellow)

Synthesis of the DNA was performed externally by Biocat GmbH (Heidelberg, Germany) and delivered in a pUC19 storage plasmid. Both sequences were restricted and integrated into the expression plasmid pPb EF1-GFP [12]. Final constructs were checked by sequencing and used for transfection of 1 × 10^6^ HEK293 cells per construct. Cells were plated in a six-well plate in cell culture medium (RPMI, Gibco, ThermoFisher Scientific, Waltham, MA, USA) and incubated at 37°C till they reached a confluency of 70%. Six microgram of each vector construct pPb EF1α-EpCAM-EGFP and pPb EF1α-NP65-EGFP, was mixed with 1 µg of helper plasmid pMB (gifted from Dr Manfred Gossen, Charite-Universitätsmedizin, Berlin, Germany) in 150 mM NaCl (150 µl final volume). pMB encoded piggybac transposase and enabled the integration of foreign DNA sequences (here fusion proteins EpCAM-EGFP and NP65-EGFP) into host genome. Piggybac transposase recognizes inverted terminal repeat (ITR) sequences which flank the foreign DNA sequences within expression vectors, cuts them and integrates into chromosomal TTAA sites (Fig. 2). In another vial, 26 µl 7.5 mM PEI solution (Sigma-Aldrich Chemie GmbH, Taufkirchen, Germany) was added and mixed with 124 µl of the vector construct containing NaCl solution. The mixture was given to the HEK293 cells in droplets, followed by an incubation of 24 h at 37°C. The transfection success was monitored via fluorescence microscopy measuring the EGFP signal. Selection of the transfected cells for a stable expression was performed by adding puromycin as antibiotic in a concentration of 2 µg/ml.

### Cultivation of HEK293 cells

Wildtype, transgenic and stable transfected HEK293 cells were cultivated in RPMI cell culture media (Gibco, ThermoFisher Scientific, Waltham, MA, USA) containing 5% newborn calf serum (Gibco, ThermoFisher Scientific, Waltham, MA, USA), 1% beta-mercaptoethanol and 1% L-glutamine at 37°C and 6.5% CO_2_.

### Expression of EpCAM and neuroplastin 65 on stable transfected cell lines

After selecting stable transfected HEK293 cells, the expression of the membrane proteins EpCAM and NP65 was checked by flow cytometry using a BD FACS ARIA III (Becton Dickinson). Wildtype HEK293 cells were used as a negative control. Cells were harvested, centrifuged for 5 min at 300 × *g* at 4°C, and washed twice with 10 ml 1× PBS. The cells were adjusted to a concentration of 1 × 10^6^ cells in 300 µl PBS. For the detection of EpCAM expression, an Alexa Flour^®^ 647-conjugated murine-anti-human EpCAM antibody (100 tests, clone VU-1D9, ab239279, Abcam, Cambridge, UK) was used. The expression of NP65 was detected with a primary rabbit-anti-human neuroplastin 65 antibody (15881-T24, SinoBiological, Beijing, China) and an Alexa Fluor^®^ 647-conjugated goat-anti-rabbit IgG H&L antibody (500 µg, ab150079, Abcam, Cambridge, Great Britain). Antibodies were incubated for 15 min at 4°C, followed by two washing steps with 10 ml 1× PBS each. The cells were centrifuged at 300 × *g* for 5 min. The pellets were resuspended in 1 ml 1× PBS. For the discrimination of dead cells, a staining with PO-PRO^TM^ (1 µg/ml, ThermoFisher Scientific, Waltham, MA, USA) was performed.

### Generation of naïve human phage display libraries

The generation of a naïve human phage display library was performed according to the protocol of Barbas *et al*. [17, 18]. Briefly, human peripheric blood mononuclear cells (PBMCs) were isolated from a buffy coat (German Red Cross). The lymphocyte containing cell pellet was washed with 20 ml MACS buffer (1× phosphate-buffered saline, 0.5% (w/v) BSA) and centrifuged at 400 × *g* for 7 min. Erythrocytes were lysed by incubating the cells in 10 ml 1× Red Blood Cell Lysis buffer (Miltenyi Biotec, Bergisch Gladbach, Germany) for 10 min at 22°C in the dark. The cells were pelleted (400 × *g*) and washed twice with 20 ml MACS buffer. The cell count was determined using a Neubauer chamber. Homogenisation of the cells was performed according to the manufacturer’s protocol with RNAPure^TM^ peqGOLD (VWR Life Science, Darmstadt, Germany). RNA was extracted using a phenol chloroform precipitation in a 1:1 ratio (300 µl PBMCs mixed with 300 µl chloroform isoamylalcohol) with an incubation time of 15 min on ice. The suspension was centrifuged (12,000 × *g*, 4°C, 7 min). The aqueous phase including the RNA was taken and mixed with the identical volume of isopropanol (pre-cooled at −20°C) and incubated for 15 min on ice. After centrifugation at 12,000 × *g* for 10 min at 4°C, the supernatant was discarded. The pellet was washed twice with 70% ethanol and dried at 55°C for 5 min. For cDNA synthesis, the pellet was dissolved in 15 µl DEPC-H_2_O and purity was determined photometrically at 260 nm.

RNA samples were transcribed to cDNA using the Proto Script ^®^ II First Strand cDNA Synthesis Kit (New England Biolabs GmbH, Ipswich, MA, USA) according to the provider’s manual.

Amplification of human single chain variable fragments (scFv) was performed as previously described [18]. First, the VL_*κ*_, VL_*λ*_, and VH chains were amplified. Primer sequences to amplify VL_*κ*_, VL_*λ*_ and VH genes were taken from [18]. Amplified fragments were analyzed by agarose gel electrophoresis for their correct sizes and cutted out of the gel for purification. With overlap extension PCR, the different heavy and light chain fragments (100 ng each) were connected with the short or the long linker fragment. After gel purification, the scFv fragments and the phagemid vector pCOMB3xSS (gifted from Scripps Research Institute, La Jolla, USA) were restricted with SfiI for 5 h at 50°C. The phagemid vector was dephosphorylated using FastAP (1 U/µl thermosensitive alkaline phosphatase, ThermoFisher Scientific, Waltham, MA, USA) upfront for 1 h at 37°C according to the manufacturer’s protocol. The restricted fragments were ligated in a ratio of 2:1 (insert to vector) for 16 h at 22°C using T4-DNA ligase (New England Biolabs GmbH, Ipswich, MA, USA) according to the manufacturer’s protocol. After heat inactivation of the ligase at 80°C for 5 min, the ligation approach was purified from an agarose gel according to the manufacturer’s protocol (NucleoSpin^®^ Gel and PCR Clean-up Kit, Macherey&Nagel, Düren, Germany) and eluted in 20 µl DEPC-H_2_O.

For phage display selection, the libraries were electroporated into *E.coli* XL1 blue using a pre-cooled 0.2 µm electroporation cuvette (BioRad GmbH, München, Germany). *E.coli* XL1 blue were thawed on ice, 100 µl were transferred into the cuvette, mixed with 5 µL of the purified ligation approach, and incubated for 1 min on ice. After the incubation, the sample was electroporated at 2.5 kV, 25 µF, 200 Ω for 5 ms. *E.coli* XL1 blue were transferred into 3 mL pre-warmed 2TY culture medium and incubated at 37°C with a shaking at 230 rpm for 1 h. After the incubation, 10 ml pre-warmed 2TY culture medium were added together with 20 µg/ml carbenicillin and 15 µg/ml tetracycline for another hour at 37°C and 230 rpm.

The size of the human naïve scFv library N5/MC was determined by diluting the transformed bacteria (1:100), 10 and 100 µL were plated on an agar plate supplemented with 50 µl carbenicillin. After incubation for 24 h at 37°C, the colonies were manually counted and the size was calculated using this formula:


$$\mathrm{librarysize}=\frac{\mathrm{numberofcolonies}\cdot \mathrm{culturevlume}\left[µl\right]}{\mathrm{platedvolume}\left[µl\right]}\cdot \mathrm{dilutionfactor}.$$

*E.coli* cells were infected with 2 ml of 1 × 10^13^ M13KO7 phages (New England Biolabs GmbH, Ipswich, MA, USA). Cell culture medium (2TY, 183 ml) was added and supplemented with 50 µg/ml carbenicillin and 10 µg/ml tetracycline. Cells were incubated at 37°C at 230 rpm for another 2 h. After this, 70 µg/ml kanamycin was added and incubated again for 16 h at 37°C and 230 rpm. The precipitation of the phages was performed by centrifugation at 3000 × *g* at 4°C for 10 min. The supernatant was mixed with $\frac{1}{5}$ of 20% w/v PEG8000/2.5 M NaCl and incubated for 30 min at 4°C. After this, the mixture was centrifuged for 25 min at 13,000 × *g* and 4°C. The pellet was dried at RT for 10 min, resuspended in 2–4 ml PBS and centrifuged for 5 min at 15,000 × *g*. The supernatant was filtered (0.22 µm), supplemented with sodium acid (1:500), and stored at 4°C for further use.

To rely on a model scFv for the establishing of the flow cytometry based selection of phages, an additional library was created with a known scFv sequence of B13DE1, a mouse monoclonal anti-fluorescein binder [11]. The DNA sequence was cloned into the phagemid vector pCOMB3xSS as described above. Monoclonal B13DE1 phages were generated using the same protocol as before and precipitated and stored for further use.

Further, a human pre-immune library was generated to investigate the use of the protocol for cell-based selection rounds with the cell line HEK293-NP65. First, three selection rounds were performed with the recombinant protein human NP65 (5360-NP-050, R&D Systems R&D Systems, Minneapolis, MN, USA) as described previously to pre-select specific binders [19, 20]. Second, the last phage preparation was taken and added to the human naïve library to mimic an immune library. Phages were precipitated and stored for further use as described above.

### Optimization experiments

Several optimization experiments were performed to establish and improve the protocol for cell- and flow cytometry-based selection round. First, the minimal necessary amount of anti-M13 phage antibody (mouse monoclonal B62 in house produced antibody) was determined. The transgenic or wildtype HEK293 cells (1 × 10^6^) were labeled with streptavidin-FITC (Miltenyi Biotec, Bergisch Gladbach, Germany). To stained cells, monoclonal B13DE1 phages (1 × 10^13^ phage particles) were added. Bound phages were detected with three amounts of B62 antibody conjugated to DyLight^®^ 650 (ab201803, Abcam, Cambridge, UK) for 15 min at 4°C [21, 22].

To mimic the real phage display selection round, mixed phages were produced from the human naïve scFv library N5/MC and monoclonal B13DE1 phages mixed in a ration 7:3. Mixed phages (1 × 10^14^ phage particles) were added to 1 × 10^6^ wildtype or transgenic HEK293 labeled with streptavidin-FITC and detected by 2.5 µg B62 antibody conjugated to DyLight^®^ 650.

To determine optimal ratio for an antigen-specific phage binding, 1 x 10^6^ transgenic HEK293 labeled with streptavidin-FITC were incubated with 1 × 10^7^, 1 × 10^10^, and 1 × 10^13^ mixed phages. Bound phages were detected with 2.5 µg B62 antibody conjugated to DyLight^®^ 650. As negative controls, streptavidin-FITC labeled transgenic HEK293 cells were incubated without phages and unlabeled cells were stained with 2.5 µg B62 antibody conjugated to DyLight^®^ 650 only.

Each incubation step was performed for 15 min at 4°C. After the incubation, three wash steps were performed with 1 ml 1× PBS and centrifugated for 5 min at 400 × *g*. As last step, cell suspensions were resuspended in 300 µl PBS and analyzed in flow cytometry (Sony SH800S Cell Sorter).

### Flow cytometry-based detection of antigen-specific phages

To select antigen-specific phages in flow cytometry, 5 × 10^14^ phages were pre-incubated with 1 × 10^6^ HEK293 wildtype cells for 15 min at 4°C. The samples were centrifuged at 400 × *g* for 5 min. The supernatant was used for incubation with 1 × 10^6^ cells of transgenic cell lines HEK293-EpCAM-EGFP and HEK293-NP65-EGFP, respectively. After 15 min at 4°C, the samples were washed three times with 1 ml PBS and centrifuged at 400 × *g* for 5 min. Binding of antigen-specific phages was detected using a mouse monoclonal anti-M13 phage antibody (mouse monoclonal, B62, 2.5 µg, in house produced antibody) conjugated to DyLight^®^ 650 as described above. After washing three times with 1× PBS, the pellets were resuspended in 300 µl 1× PBS and analyzed in flow cytometry (Sony SH800S Cell Sorter). As negative control, all three cell lines were stained in the same manner with the B62 antibody but without phages. Positively sorted phages were used to re-infect *E.coli* XL1 blue cells (2 ml at OD_600_=1.0) for 30 min at room temperature (RT). Cultivation was performed in 6 ml 2TY medium supplemented with 20 µg/ml carbenicillin and 10 µg/ml tetracycline. After 1 h at 37°C at 230 rpm, 50 µg/ml carbenicillin were added to the culture and incubated for another hour.

### Polyclonal antigen-specific phage ELISA

To test for antigen-specificity, precipitated phages (diluted 1:5 and 1:50) were incubated on microtiter plates coated with 5 µg/ml recombinant extracellular domain of EpCAM (ab15338, Abcam, Cambridge, UK) or 2.5 µg/mL recombinant human NP65 for 90 min in a humid chamber. Free binding sites were blocked with 3% BSA/1× PBS (100 µl/well) for 45 min at RT. Detection of bound phages was performed using a HRP-conjugated anti-M13 phage antibody (mouse monoclonal, B62, 1:7000, in house produced antibody) for 45 min in a humid chamber. Between all steps, the plates were washed 10 times with tap water. Enzyme-substrate reaction was induced by adding 50 µl substrate solution (0.1 M H_2_PO_4_, 0.1% (w/v) urea/H_2_O_2_, 1.2 mg 3,3’,5,5 tetramethylbenzidine in ethanol) to the wells. The plates were incubated for 8 min in the dark. The reaction was stopped adding 1 M H_2_SO_4_. Optical densities were measured at OD 450 nm with a reference wavelength of 620 nm.

### Monoclonal antigen-specific phage ELISA

After one cell-based selection round, individual clones were randomly selected to produce monoclonal phage particles as described in [23]. Next, the monoclonal phage particles were tested for the antigen-specificity with recombinant extracellular domains of EpCAM or NP65. 5 µg/ml of EpCAM or 2 µg/ml of NP-65 were coated over night at 4°C. Additionally, the anti-M13 phage antibody (B62), blocking solutions 3% BSA/1× PBS for ELISA with EpCAM or 1% casein/1× PBS for ELISA with NP65 were coated to detect the phage particles in monoclonal phage preparations and to test the cross-reactivity. Free binding sites were blocked with 3% BSA/1× PBS or 1% casein/1× PBS (100 µl/well) for 45 min at RT. Monoclonal phage preparations (50 µl/well) were added undiluted to coated wells. Detection of bound phages was performed using a HRP-conjugated anti-M13 phage antibody (mouse monoclonal, B62, 1:7000) for 45 min in a humid chamber. Between all steps, the plates were washed 10 times with tap water. Enzyme-substrate reaction was induced by adding 50 µl substrate solution (0.1 M H_2_PO_4_, 0.1% (w/v) urea/H_2_O_2_, 1.2 mg 3,3’,5,5 tetramethylbenzidine in ethanol) to the wells. The plates were incubated for 8 min in the dark. The reaction was stopped adding 1 M H_2_SO_4_. Optical densities were measured at OD 450 nm with a reference wavelength of 620 nm.

### Cloning of antigen specific sequences for expression of human IgG4 full length antibodies

Antigen-specific monoclonal phages were sequenced and used for the assembly of full length human IgG4 antibodies. The in house-generated expression vector was encoded for hinge, CH2 and CH3 domains of human IgG4 under the control of CMV promoter. Selected scFv fragments were amplified and integrated into the expression vector using In Fusion^®^ HD Cloning Kit according to the manufacturer’s protocol (Takara Bio INC., Kusatsu, Japan). Transformation of 50 µl *E.coli* HST08 Stellar cells (Takara Bio INC., Kusatsu, Japan) with 5 µL of the ligation mix was performed for 30 min on ice. Afterward, the cells were heated at 42°C for 42 s. The transformation sample was filled up to 1 ml with SOC medium and incubated on ice for 5 min, followed by a 1 h incubation at 37°C and 230 rpm. Transformed bacteria cells were pelleted at 4000 × *g* for 4 min. The pellet was resuspended in 200 µl SOC medium and plated on agar plates supplemented with 50 µg/ml carbenicillin, followed by an incubation at 37°C overnight. Plasmids were sequenced (LGC Genomics GmbH, Berlin, Germany) for confirmation and used for the expression of recombinant human full-length antibodies in mammalian cells.

### Expression of recombinant human full-length antibodies of IgG4 isotype

Expression of full-length antibodies was performed with high density Expi293F^TM^-cells (Gibco ThermoFisher Scientific, Waltham, MA, USA) according to the manufacturer’s protocol.

Cell culture supernatants (200 µl from each construct) were collected during cultivation of the cells and centrifuged at 3200 × *g* for 20 min. Antibody production was proved by ELISA with an anti-human IgG antibody (2.5 µg/ml, Sigma-Aldrich, St Louis, MO, USA) as capture and an HRP-conjugated anti-human IgG antibody (1:5000, Sigma-Aldrich, St Louis, MO, USA) as detector. Culture supernatants containing produced antibodies were added to the wells (50 µl/well) and incubated for 45 min at RT. Free binding sites were blocked upfront with PBS/3% BSA (100 µl/well) for 45 min at RT in a humid chamber. In between all steps, the wells were washed 10 times with tap water. Enzyme-substrate reaction was induced by adding 50 µl substrate solution (0.1 M H_2_PO_4_, 0.1% (w/v) urea/H_2_O_2_, 1.2 mg 3,3’,5,5 tetramethylbenzidine in ethanol) to the wells and the reaction was stopped with 1 M H_2_SO_4_ after 8 min in the dark. Optical densities were measured at 450 nm with a reference wavelength at 620 nm.

### Purification of antibodies

Antibodies were purified by affinity chromatography using a HiTrap Protein G HP column (Äkta pure™, Cytiva, Marlborough, MA, USA) as described previously [24]. Briefly, culture supernatants were mixed with binding buffer (20 mM sodium phosphate buffer pH 7.0) in a ratio of 1:1. After equilibration of the column with 30 ml binding buffer, the supernatants were loaded with a flow rate of 5 ml/min. Unspecific proteins were washed from the column with 50 ml of binding buffer. Elution of the antibodies took place by a pH shift from 7.0 to 2.0. Eluates were collected in 1.5 ml fractions and immediately neutralized by adding 150 µl neutralization buffer (3.5 M Tris-HCl buffer pH 9.1). For further processing, purified antibody fractions were dialyzed against 1× PBS, the absorption was measured at 280 nm, and the concentration of the antibody was calculated with the corresponding extinction coefficient determined according to amino acid sequences of antibody.

### Characterization of recombinant IgG4 antibodies

Purified antibodies were characterized by SDS PAGE, Western Blot, ELISA, and flow cytometry. SDS PAGEs were performed with 10% polyacrylamide gels according to the standard protocol published in Laemmli [25]. Antibody samples (1–3 µg) were mixed with 6× reduced sample buffer and adjusted to 25 µl with tap water. Afterward, the samples were denatured for 10 min at 70°C. The separation of the protein bands was performed at 160 V for 45–60 min. Gels were stained with Coomassie and documented using a Molecular Imager (Gel Doc ™ XR). To verify that the recombinantly produced and purified IgG4 antibodies were expressed with the corresponding Fcγ part, a human Fcγ specific antibody conjugated to HRP was used (1:5000, A0170, Sigma-Aldrich, St Louis, MO, USA) in a Western Blot. Free binding spots were saturated with 15 ml blocking solution (ROTIBlock, Carl Roth GmbH, Karlsruhe, Germany) for 30 min at RT. After washing three times with 15 ml 1× TBS/0.05% Tween20 and one time with 1× TBS, protein bands were stained with Clarity ™ Western Blot ECL (BioRad GmbH, München, Germany) substrate solution according to the manufacturers protocol and incubated for 1 min in the dark. Chemiluminescent signals were detected in a Molecular Imager Gel Doc ™XR.

Antigen specificity was proven by ELISA. Antigen was coated to the solid phase (5 µg/ml human recombinant EpCAM, 50 µl/well) and incubated over night at 4°C in a humid chamber. After a washing step (three times with tap water), the wells were blocked with PBS/3% BSA (100 µl/well) for 30 min at RT. Human recombinant antibodies were diluted from 0 till 50 µg/ml in 1:2 steps with the blocking solution. The commercial mouse anti-EpCAM antibody (HEA-125, 130-113-268, Miltenyi Biotec, Bergisch Gladbach, Germany) was diluted from 0 to 1000 ng/µl with blocking solution. Bound human antibodies were detected with HRP-conjugated anti-human IgG antibody (1:5000, Sigma-Aldrich, St Louis, MO, USA) and HEA-125 with HRP-conjugated goat anti-mouse antibody (1:5000, Dianova, Hamburg, Germany) for 45 min at RT in a humid chamber. In between all steps, the wells were washed 10 times with tap water. Enzyme–substrate reaction was induced by adding 50 µl substrate solution (0.1 M H_2_PO_4_, 0.1% (w/v) urea/H_2_O_2_, 1.2 mg 3,3’,5,5 tetramethylbenzidine in ethanol) to the wells and the reaction was stopped with 1 M H_2_SO_4_ after 8 min in the dark. Optical densities were measured at 450 nm with a reference wavelength at 620 nm.

Antigen specificity was also investigated by flow cytometry. Respectively, 1 × 10^6^ wildtype HEK293 cells and HEK293 cell line expressing the fusion protein EpCAM-EGFP (HEK293-EpCAM-EGFP) were incubated with 4.5, 15, and 22.5 µg of human recombinant D7-IgG4 antibody for 15 min at 4°C. Bound antibodies were detected with goat anti-human IgG4 antibody conjugated with AlexaFluor^®^ 647 (Sigma-Aldrich, St Louis, MO, USA) for 15 min at 4°C. As negative controls, cells were only incubated with the detection antibody. Between incubations, three washing steps were performed with 10 ml 1x PBS and centrifugation for 5 min at 300 × *g*. After washing three times with PBS, the pellets were resuspended in 300 µl PBS and analyzed in flow cytometry (Sony SH800S Cell Sorter).

## Results

### Over expressing EpCAM and NP65 cell lines

**Figure 3:**
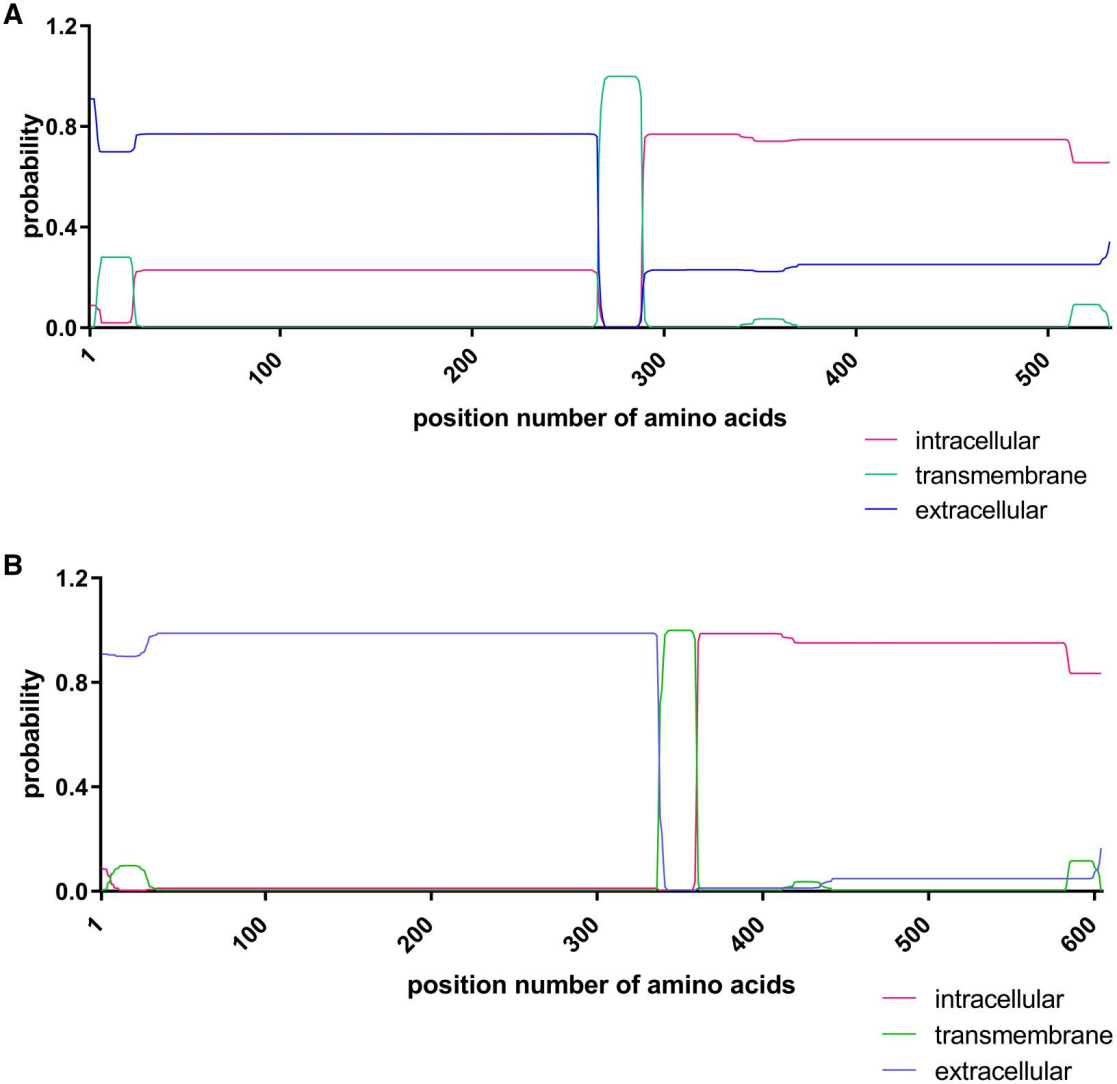
In silico prediction for the localization of domains of the fusion membrane proteins EpCAM-EGFP (**A**) and NP65-EGFP (**B**) using the online tool TMHMM-2.0 (DTU Health Tech). The probability for the localization of domain (intracellular, transmembrane or extracellular) was calculated at each amino acid position and presented as a graph

To ensure the right localization of domains for both fusion proteins, an *in silico* modeling of transmembrane helices was performed using DTY Health Tech, TMHMM-2.0. The analysis for EpCAM-EGFP fusion showed that amino acids (aa) 266 till 288 were predicted as transmembrane part whereas aa 24 till 265 were predicted as extracellular (Fig. 3A). For the NP65-EGFP fusion the same *in silico* prediction model was used showing that aa 29 till 337 were predicted as extracellular and aa 338 till 359 as transmembrane (Fig. 3B). The EGFP domains of EpCAM (aa 289 till 532) and of NP65 (aa 360 till 604) referred to the intracellular part which was exchanged instead of the intracellular domain of EpCAM or NP65. The *in silico* analysis for both fusion proteins indicated the right localization of extracellular domains of EpCAM and NP65, thus the sequences could be used to generate overexpressing EpCAM and NP65 stable cell lines.

**Figure 4:**
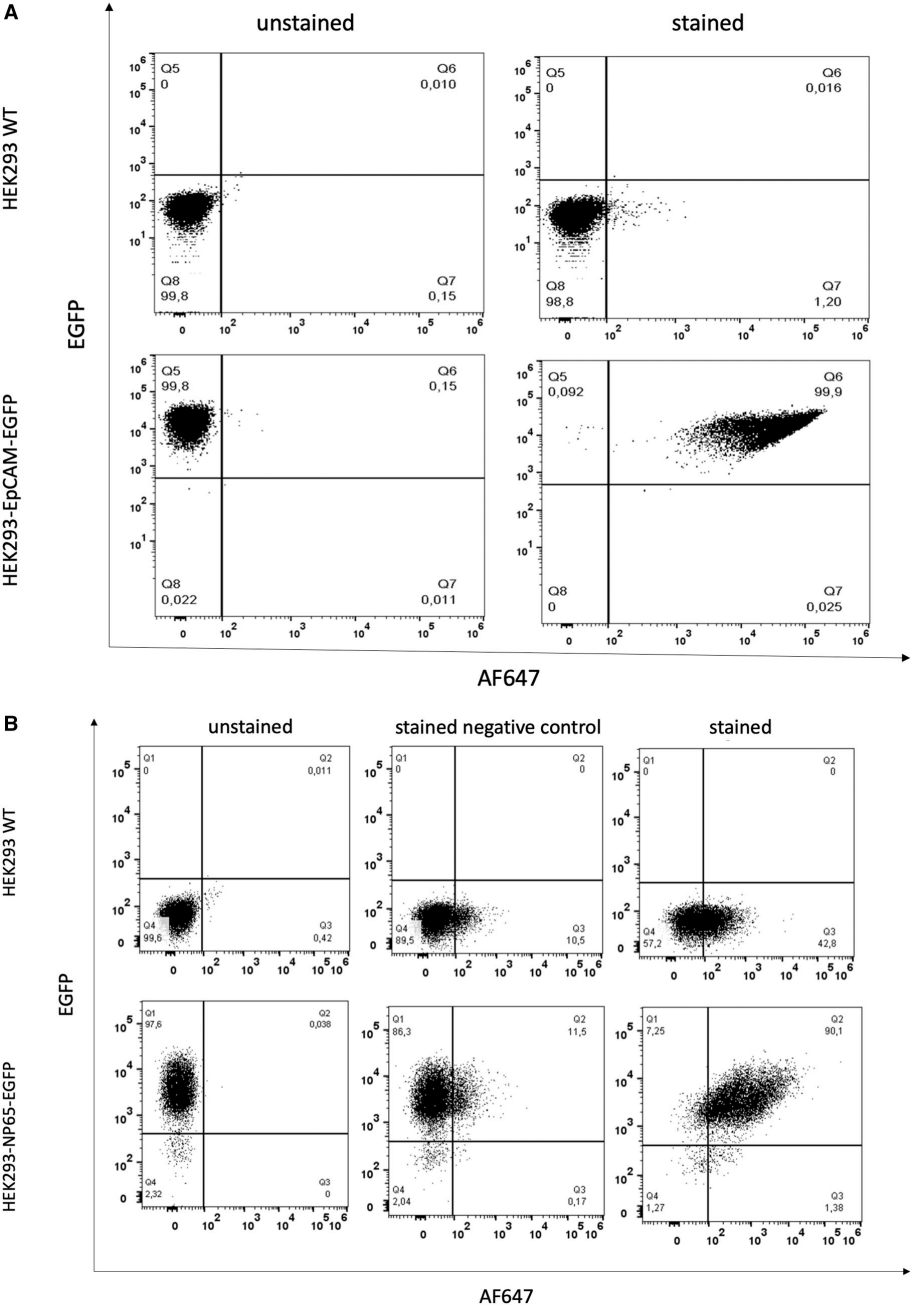
Flow cytometric analysis of generated cell line expressing the extracellular domains of EpCAM (**A**) and NP65 (**B**). Fluorescence intensities for live cell population of stable cell lines HEK293-EpCAM-EGFP (A), HEK293-NP65-EGFP (B), and of wildtype HEK293 cells (A, B) were presented in the EGFP and AlexaFluor^®^647 (AF647) channel. The expression of EpCAM extracellular domain was detected with anti-EpCAM antibody conjugated with AF647. The expression of NP65 extracellular domain was detected with primary rabbit anti-NP65 antibody and secondary goat anti-rabbit antibody conjugated with AF647. As negative control the wildtype HEK293 cells were used

After transfection of HEK293 cells with the corresponding vectors, pEF1α-EpCAM-EGFP and pEF1α-NP65-EGFP, a selection with puromycin was performed to establish stable transfected cell clones. Extracellular domains of EpCAM and NP65 were co-expressed as fusion proteins with intracellular EGFP showing flow cytometric signal of 100% for both proteins (Fig. 4). In addition, the expression of EpCAM and NP65 on the cell surface was checked using specific antibodies. For EpCAM, the specific signal was confirmed with 99.9% (Fig. 4A). The results for NP65 showed a specific staining of 90.1% (Fig. 4B). Non-transfected wildtype HEK293 cells were used as negative controls. Both over expressing cell lines showed a significant shift of the EGFP signal by 99.8% (EpCAM-EGFP) and 97.6% (NP65-EGFP). 42.8% of wildtype HEK293 were positively stained with the primary rabbit anti-NP65 antibody and detected by the secondary antibody. However, this cell population is EGFP-negative concluding that the wildtype HEK293 were not expressing the fusion protein NP65-EGFP, but the primary rabbit anti-NP65 antibody could possibly cross react with some cell surface proteins of wildtype HEK293.

### Flow cytometric selection of human naïve phage display libraries

For establishing an antigen-specific selection of phage libraries with a cell-based system, a human naïve scFv phage library was created from five human donors named N5/MC. VL and VH domains within this library were connected with a long linker containing seventeen amino acids. The library size was determined with 1.5 × 10^9^.

**Figure 5:**
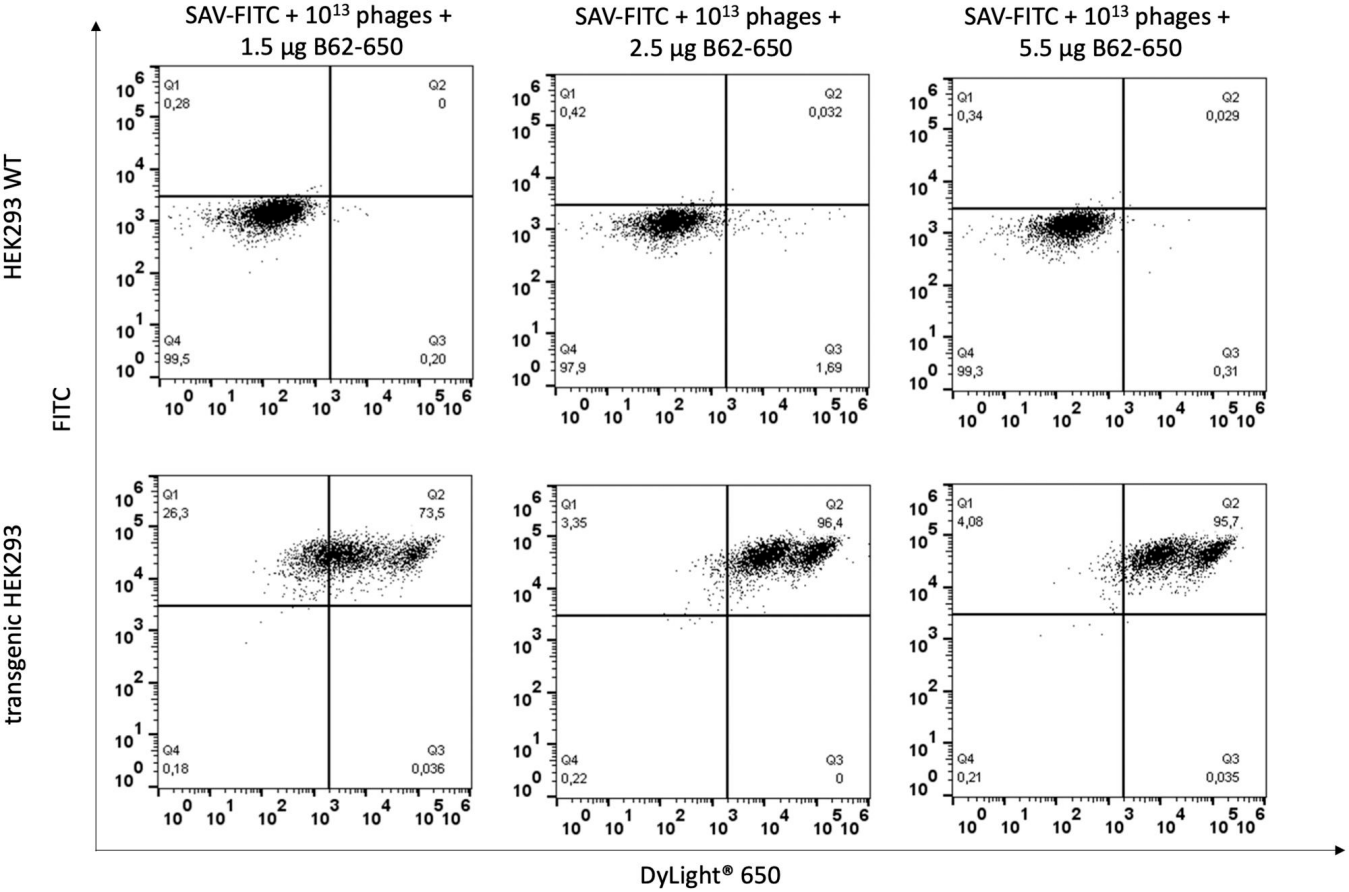
Flow cytometric analysis to determine the necessary amount of anti-phage antibody (B62) conjugated with a fluorescent dye DyLight^®^ 650 (B62-650) for further detection of antigen-specific phage binding. Wildtype HEK293 cells and transgenic HEK293 cells were labeled with streptavidin-FITC. Monoclonal phage library with murine anti-FITC B13DE1 binders were added to the cells. The phage binding was detected with three amount of anti-phage antibody conjugated with DyLight^®^ 650. Fluorescence intensities were presented in FITC and DyLight^®^ 650 channels

To first define the minimal necessary amount of murine anti-M13 phage antibody (B62) for detecting the antigen-specific phage binding, the monoclonal phage library presenting murine anti-FITC B13DE1-scFv fragments was generated. This phage library was incubated with the FITC-streptavidin labeled transgenic HEK293 cells (Fig. 5). The bound phage particles were detected with three concentrations of the anti-phage antibody conjugated with DyLight^®^ 650 (B62-650). Cytometric signals of 96.4% and 95.7% were achieved for 2.5 and 5.5 µg, respectively. As negative control, wildtype HEK293 cells were labeled with FITC-streptavidin and incubated with the monoclonal phage library. After detection with the anti-phage antibody no signal was measured.

**Figure 6:**
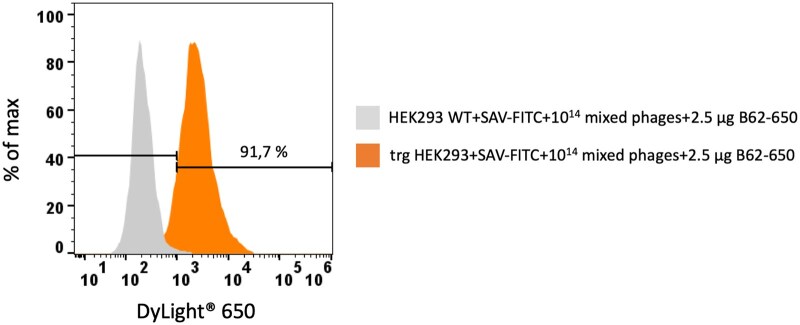
Flow cytometric analysis of antigen-specific phage binding. The polyclonal phage library including the well characterized anti-FITC scFv B13DE1 binder was incubated with streptavidin-FITC labeled transgenic HEK293 cells and wildtype HEK-293 cells as negative control. The FITC-specific phage binding was detected with 2.5 µg anti-M13 phage antibody (B62) conjugated with DyLight^®^ 650

To evaluate the specificity of the binding events and to calculate the optimal amount of phages for flow cytometric selection, the phage library N5/MC was spiked with a phage library of monoclonal phages expressing the well characterized murine anti-FITC scFv fragment B13DE1 [11]. The mixed approach was screened on transgenic HEK293 cells labeled with FITC-streptavidin. As shown in Fig. 6, the antigen-specific binding of phages could be detected by 91.7%. An unspecific binding to wildtype HEK293 was not observed.

**Figure 7:**
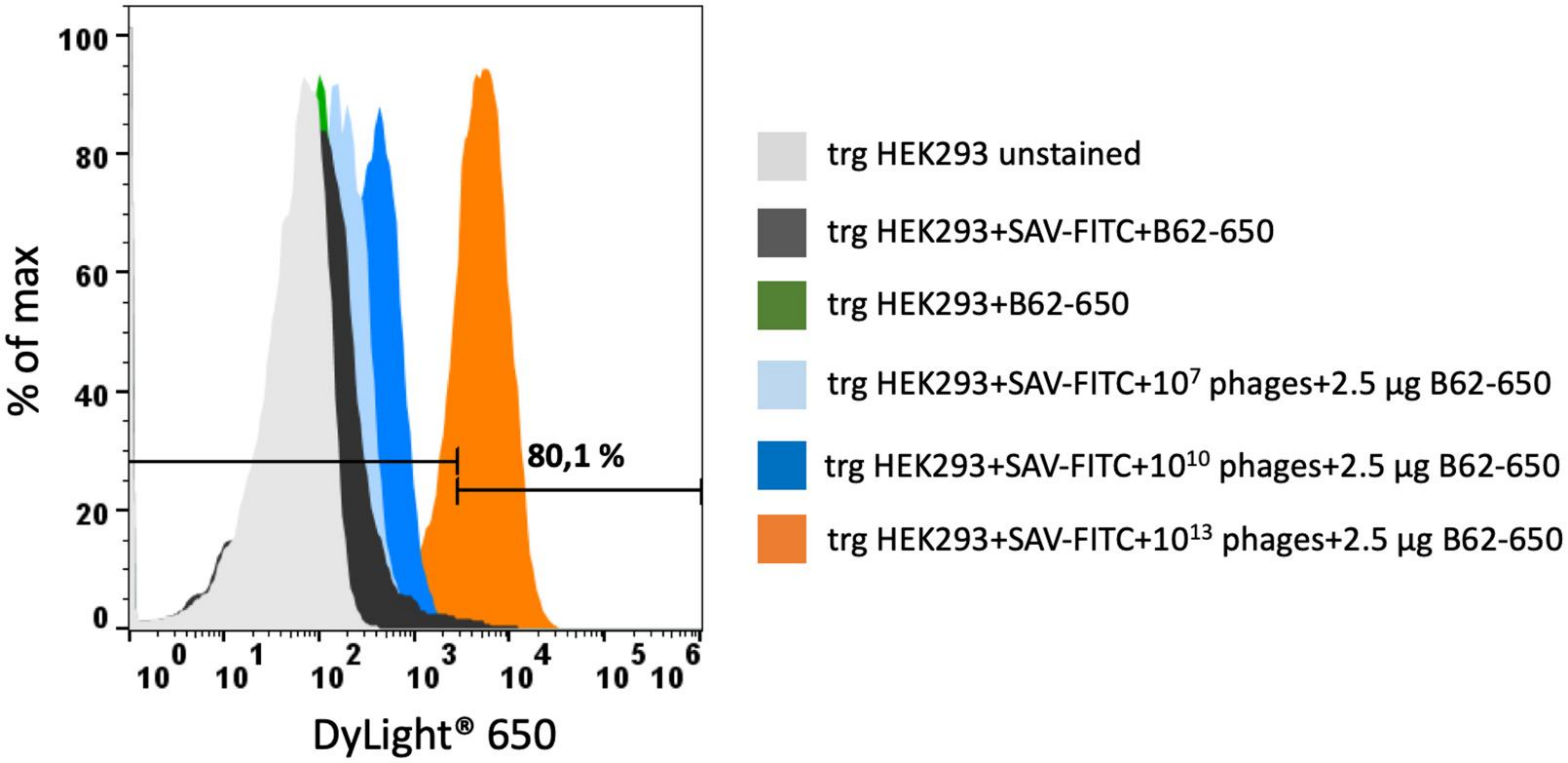
Determination of the optimal ratio for an antigen-specific phage binding. Different amount of phage particles (10^7^, 10^10^, 10^13^) from the polyclonal phage library including the well characterized anti-FITC scFv B13DE1 binder were incubated with FITC-streptavidin labeled transgenic (trg) HEK293 cells. The FITC-specific phage binding was detected with 2.5 µg anti-phage antibody conjugated with DyLight^®^ 650

Next, different concentrations of phage particles were titrated to determine the optimal ratio for an antigen-specific selection (Fig. 7). At least, with 1 × 10^13^ phage particles a specific signal of 80% could be achieved. For further selection, also a higher concentration than 1 × 10^13^ phage particles can be used as showed in Fig. 6, to reach higher cytometric signals increasing the possibility to identify appropriate binders.

The optimized and improved protocol for the cell-based selection round was accomplished on the basis of preliminary optimizations experiments as summarized in Fig. 1. First, the amount of anti-M13 phage antibody was determined to detect the antigen-specific phage binding. Second, it was shown that the antigen-specific phage binding from the polyclonal phage library could be measured in the flow cytometry. Third, the optimal ratio of phage particles from the phage library was determined to ensure the appropriate amount of specific binders within the polyclonal phage library.

Afterwards, 5 × 10^14^ phage particles from the generated phage library were pre-incubated with 1 × 10^6^ wildtype cells to eliminate unspecific binders. Next, the phage particles from the library were incubated with the generated cell line expressing the target membrane protein. After several washing steps, the phage binding was detected with 2.5 µg anti-phage antibody conjugated with a fluorescent dye DyLight^®^650. As negative control and to set the sorting gate, wildtype cells with unspecific bound phage particles were also stained with 2.5 µg detection antibody. The sorted phage particles are sorted according to the negative control and enriched for further investigations.

**Figure 8:**
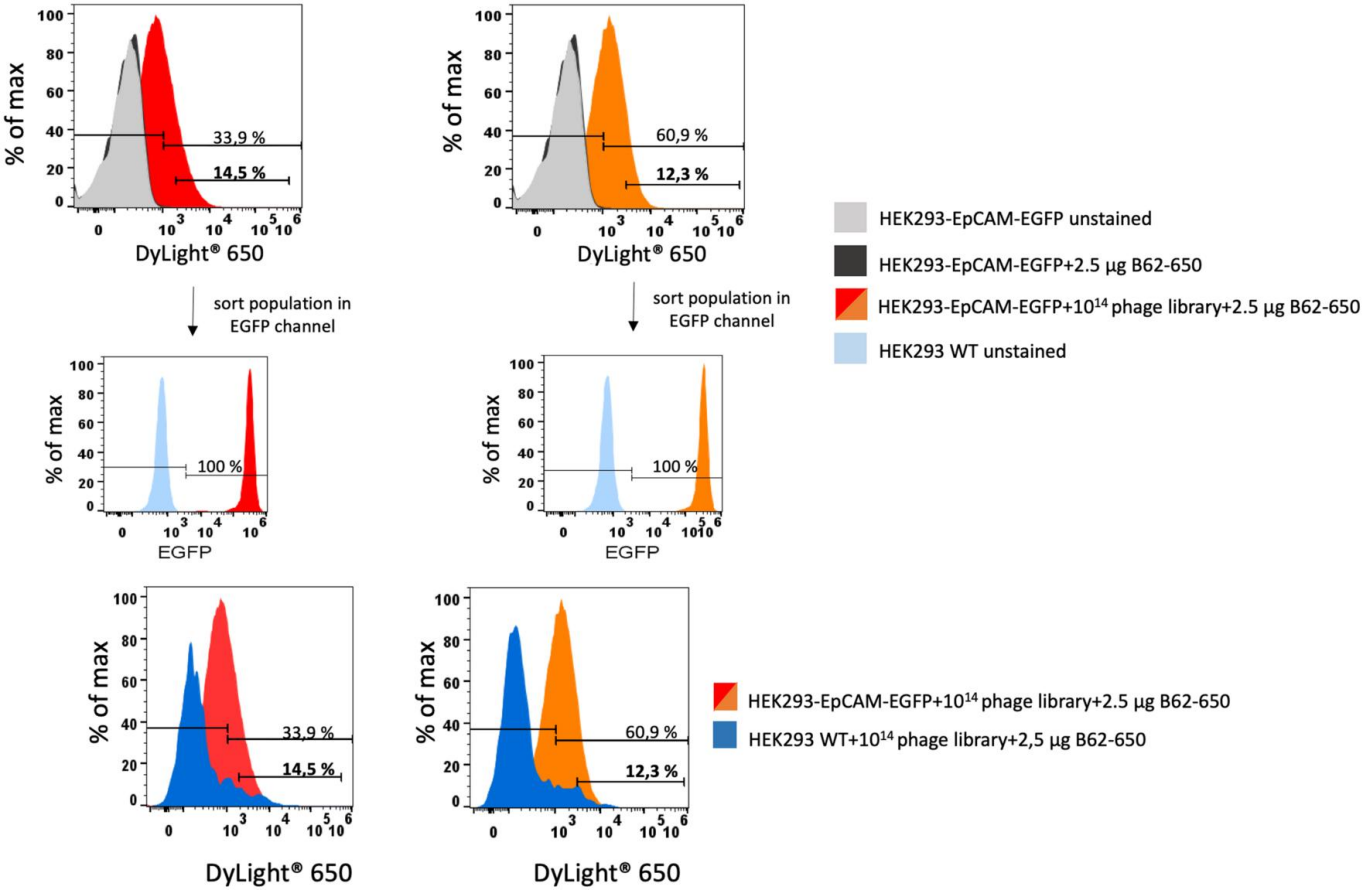
Cell-based selection round with the stable cell line HEK-293-EpCAM-EGFP. About 5 × 10^14^ phage particles from the human naïve scFv phage library were pre-incubated with 1 × 10^6^ wildtype HEK293 cells. Unbound phage particles were added to 1 × 10^6^ cells expressing the extracellular domain of EpCAM as fusion protein with EGFP (intracellular domain). Both approaches were incubated with an anti-phage antibody conjugated with DyLight^®^ 650. Fluorescence intensities for those cell populations were presented as histograms in DyLight^®^ 650 channel. The sorting gate was set up according to the negative control. Moreover, sort populations were presented in the EGFP channel for cross checking the expression of EpCAM extracellular domain

For the flow cytometric selection rounds, 5 × 10^14^ phage particles were incubated with 1 × 10^6^ HEK293-EpCAM-EGFP or HEK293-NP65-EGFP using the optimized selection protocol. Figure 8 shows the staining results for EpCAM with 33.9% specific binding in the first attempt and 60% in the second attempt. Because of higher background signals to wildtype HEK293 cells and to avoid sorting of unspecific phages, the sorting gate was set at 10^3^ and respectively, 14.5% and 12.3% of the phages were sorted. The population of sorted phages showed a cytometric signal of 100% in the EGFP channel, meaning that all of the sorted cell expressed EpCAM on the surface.

**Figure 9:**
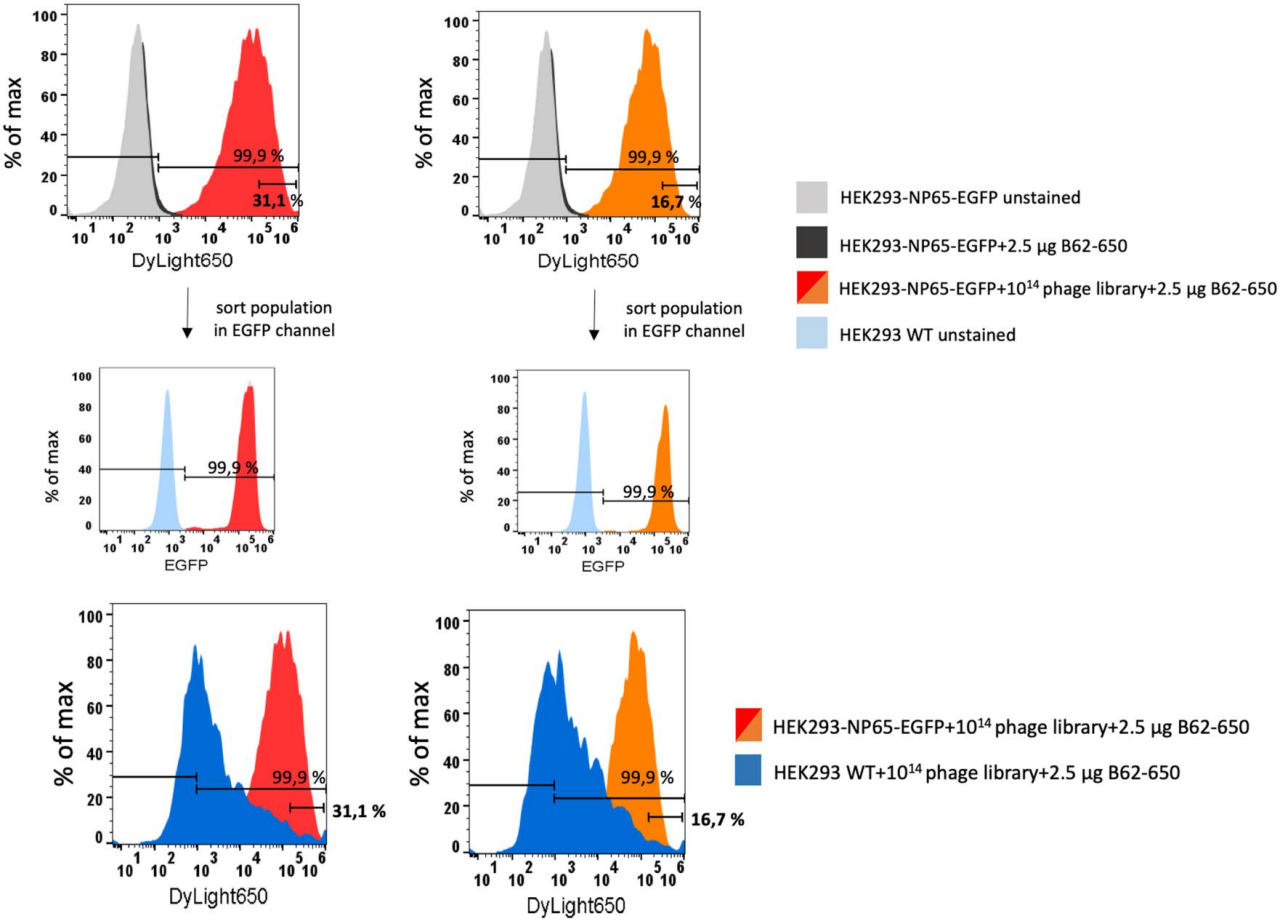
Cell-based selection round with the stable cell line HEK293-NP65-EGFP. 5 × 10^14^ phage particles from the human naïve scFv phage library were pre-incubated with 1 × 10^6^ wildtype HEK293 cells. Unbound phage particles were added to 1 × 10^6^ cells expressing the extracellular domain of NP-65 as fusion protein with EGFP (intracellular domain). Both approaches were incubated with an anti-phage antibody conjugated with DyLight^®^ 650. Fluorescence intensities for those cell populations were presented as histograms in DyLight^®^ 650 channel. The sorting gate was set up according to the negative control. Moreover, sort populations were presented in the EGFP channel for cross checking the expression of NP65 extracellular domain

Next, the cell-based selection was performed with the HEK293-NP65-EGFP cell line to show the ability of its use with another antigen. Thus, 31.1% of bound phages were sorted in the first round and 16.7% in the second round. The sorting gate was set at 10^5^ because of the background signal for wildtype HEK293 cells (Fig. 9). The specific signal for EGFP was at 100% for both generated cell lines.

**Figure 10:**
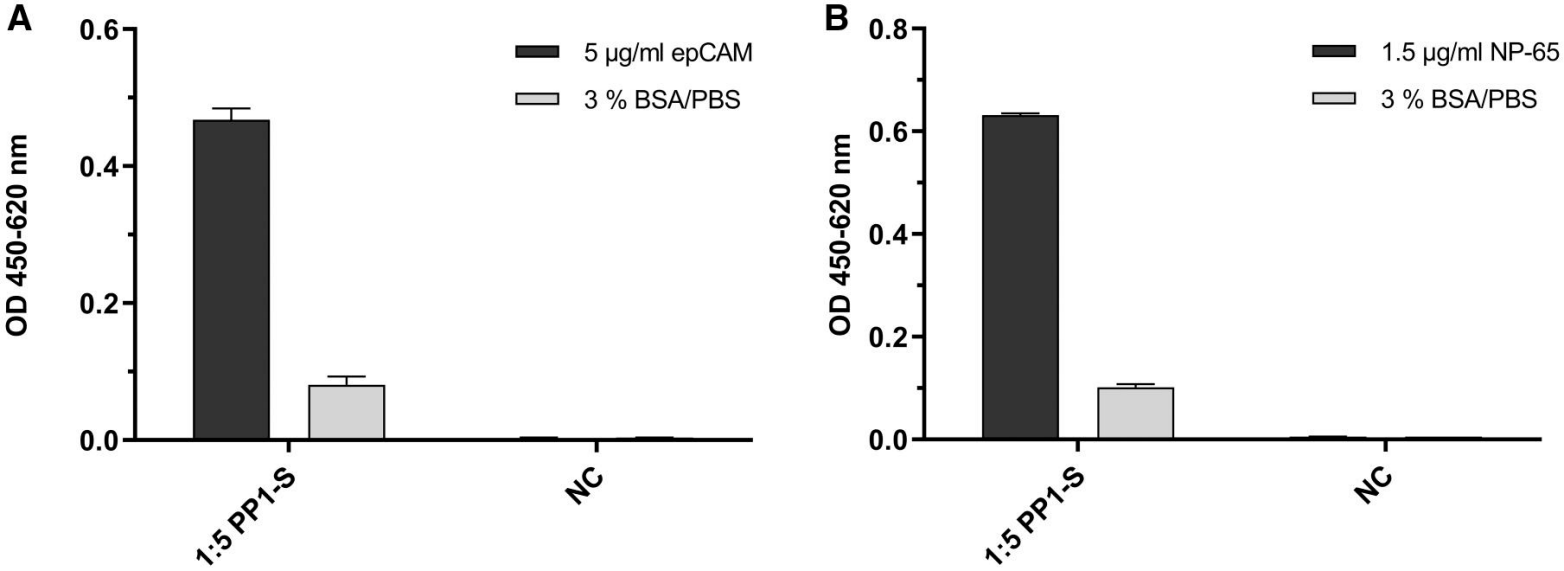
Polyclonal phage ELISA to investigate the EpCAM and NP65 specificity of sorted phage preparations (PP1-S). The recombinant extracellular domain of EpCAM (5 µg/ml) (**A**) and of NP65 (**B**) were coated onto the solid phase. As negative control, the blocking solution (3% BSA in 1× PBS) was used instead of recombinant antigen. Sorted phage preparation were diluted 1:5 with the blocking solution. Bound phages were detected with HRP-labeled anti-phage antibody (1:7000). No phage dilution was used in the negative control (NC). Average values were calculated and presented with standard deviations (*n* = 2)

The sorted phage fractions (PP1-S) were collected and used for the generation of monoclonal phages. Further, the polyclonal fractions were tested in ELISA on recombinant human EpCAM (Fig. 10A) with a specific signal of 0.45 and on recombinant human NP65 (Fig. 10B) with a specific signal of 0.6.

### Generation of monoclonal phage clones

**Figure 11:**
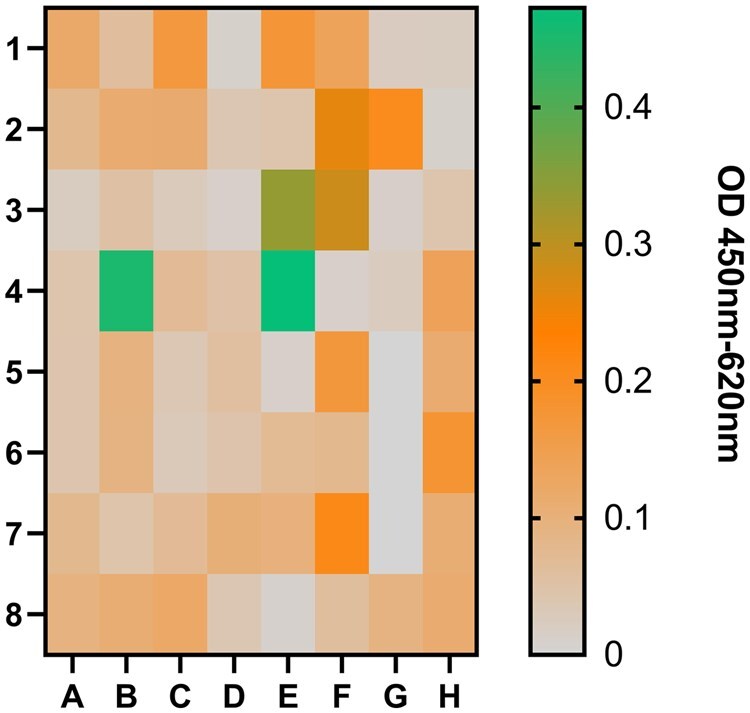
Monoclonal phage-ELISA to investigate the EpCAM-specificity of monoclonal phage preparations representing scFv fragments on their surfaces. The recombinant extracellular domain of EpCAM (5 µg/ml) were coated onto the solid phase. The monoclonal anti-M13 phage antibody (B62, 5 µg/ml) were also coated to detect the presence of monoclonal phages in preparation. As negative control, the blocking solution (3% BSA in 1× PBS) was used instead of recombinant antigen. Monoclonal phage preparations were added undiluted to the coated wells. Bound phages were detected with HRP-labeled anti-phage antibody (1:7000). Average values were calculated (*n* = 2) for EpCAM and blocking solution. Blocking solutions signals were subtracted from EpCAM signals. Antigen-specific signals were normalized to B62-signals. Each signal in the presented heat map corresponded to the well of ELISA plate

The flow cytometric selection of antigen-specific phages led to 62 insert-positive clones for EpCAM. From these clones, 5 (8%) showed a positive ELISA signal for EpCAM (scFv-L-B2,- C2, -F5, -H5 and VH-D7). Clones B4, E3 and E4 showing very high EpCAM signals exhibited high cross reactivity signals to the His-tag (data not presented) and were excluded for further examinations. The output was compared to a standard phage display panning using the recombinant extracellular domain of EpCAM with 392 clones in total and 32% insert-positive ones (data not shown). From these 32% insert-positive clones only two clones showed a specific ELISA signal on EpCAM (1.5%) (Fig. 11).

Sequence analyses of selected clones after the cell-based selection round showed various amino acid composition within CDRs in VH chains (Tables 1 and 2). VH chains of selected clones belonged mainly to the VH III subgroup which is the one of the most represent subgroup together with VH II [26–28]. Lengths of VH chains were in the range between eight and eleven amino acids.

**Table 1.** Amino acid sequences of VH chain CDRs.

	VH chain			
	subgroup			
		CDR-H1	CDR-H2	CDR-H3
**scFv-L-B2**	III	NFWMS	NINEDGGEKYYADSVKG	ELIGGLNFFEH
**scFv-L-C2**	III	DYAMH	GITWNNDKIDYADSVKG	GRTHNFLTGYLDH
**VH-D7**	III	AYDMA	YISNSGGIVYYADSVKG	DREGDKRGDH
**scFv-L-F5**	II	GYYWS	EINHSGSTNYNPSLKS	GGVTLDY
**scFv-L-H5**	III	DRYLD	RMRDKANGHPTEYAASVKG	DFLGTGDY

**Table 2.** Amino acid sequences of VL chain CDRs.

	VL chain			
	subgroup			
		CDR-L1	CDR-L2	CDR-L3
**scFv-L-B2**	Kappa III	RTSQSVSNSHFA	GSSYRAT	QVYGTSWWT
**scFv-L-C2**	Lambda II	TGSSSDVGAYKYVS	EVNNRPS	SSHTSIPSVV
**VH-D7**	–	–	–	–
**scFv-L-F5**	Kappa I	RASQTIYNDLI	GASTLQS	QQSYVTPIT
**scFv-L-H5**	Kappa III	RASESVYNTYLA	GASNRAT	QQYGSSPIT

Two of the identified clones were paired with VL chains belonging to the subgroup kappa III. The pairing between VH III and VL kappa III is rare [29].

**Figure 12:**
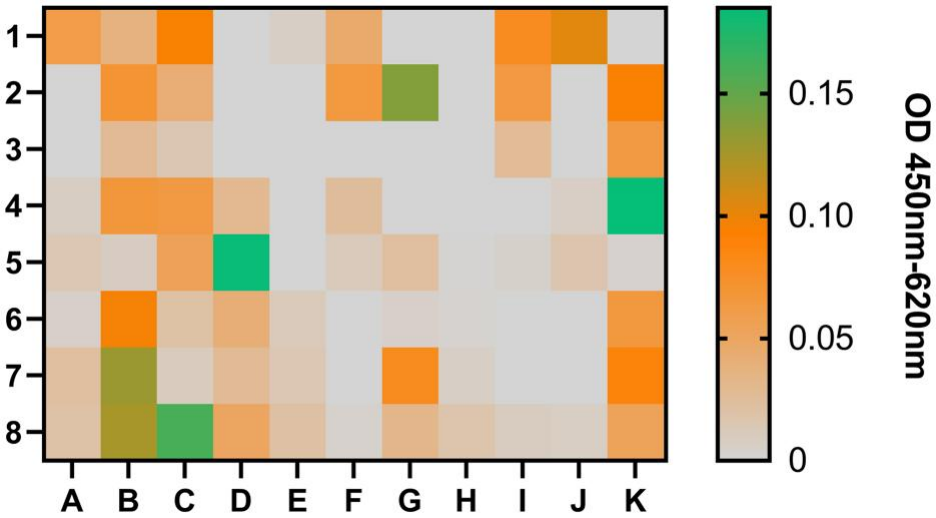
Monoclonal phage-ELISA to investigate the NP65-specificity of monoclonal phage preparations. The recombinant extracellular domain of NP65 (2 µg/ml) was coated onto the solid phase. The monoclonal anti-M13 phage antibody (B62, 5 µg/ml) was coated to detect the presence of monoclonal phages in preparation. As negative control, the blocking solution (1% casein in 1x PBS) was used instead of recombinant antigen. Monoclonal phage preparations (undiluted) were added to the coated wells. Bound phages were detected with HRP-labeled anti-phage antibody (1:7000). Antigen-specific signals were normalized to B62-signals. Each signal in the presented heat map corresponded to the well of ELISA plate

From 88 NP65 specific clones, 7 (7.9%) showed a positive ELISA signal for NP65 (scFv-L-B7, -B8, -C8, -D5, -G2, -J1 and -K4) as presented in Fig. 12.

### Characterization of selected antibodies

**Figure 13:**
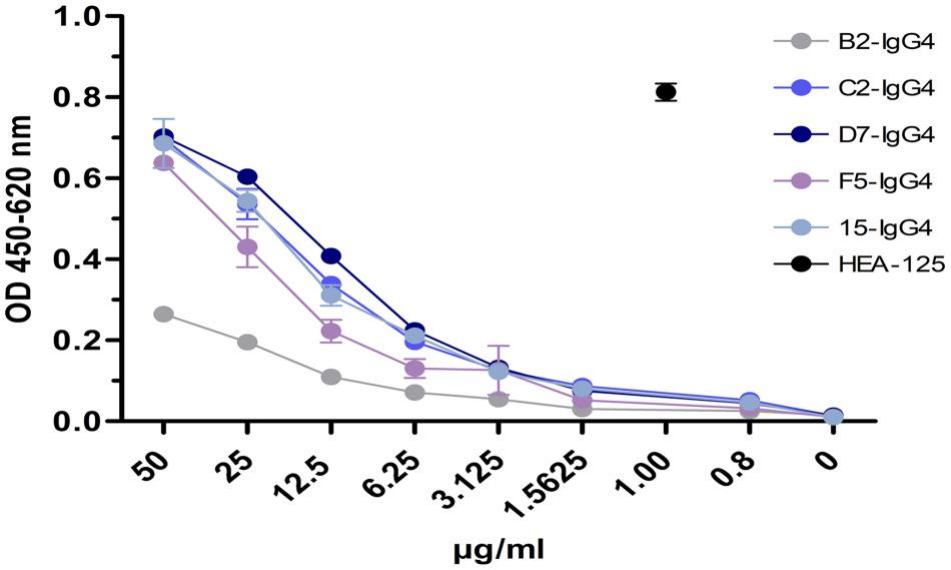
Direct immunoassay to examine the EpCAM specificity of monoclonal human recombinant antibodies and of the commercial positive control antibody HEA-125. Recombinant extracellular domain of EpCAM was immobilized onto the solid phase and human antibodies were added in different concentrations 0–50 µg/ml. The signal of the positive control antibody HEA125 is shown as black dot at a concentration of 1 µg/ml. Bound antibodies were detected with an anti-human IgG4 antibody and an anti-mouse antibody for HEA-125, both conjugated with horseradish peroxidase (HRP). As negative control, the blocking solution (3% BSA in 1× PBS) was used instead of recombinant antigen. Average values were calculated for each concentration and presented in a graph with standard deviation (*n* = 3)

For the expression of full length EpCAM specific antibodies, five different candidates were expressed with an IgG4 scaffold and tested in an indirect ELISA with coated EpCAM (Fig. 13). The purified antibodies were tested in a 1:2 dilution series from 50 µg/mL and compared to a commercial positive control antibody (HEA-125). In comparison to the control antibody, the signals generated by the newly generated antibody candidates were 50fold lower than the control antibody.

**Figure 14:**
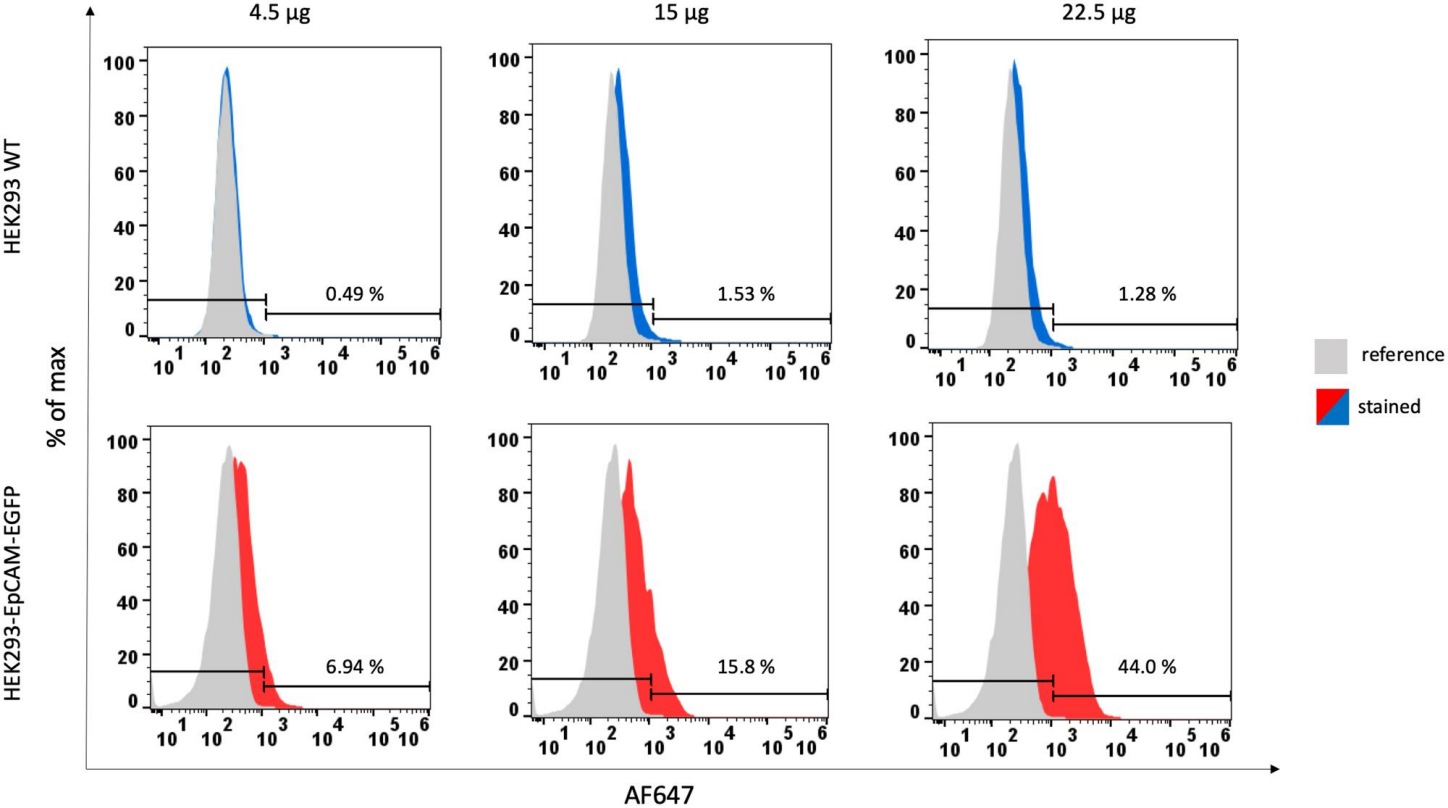
Flow cytometric analysis to investigate the EpCAM specificity of the human recombinant D7-IgG4 antibody. The cell line expressing the extracellular domain of EpCAM (HEK293-EpCAM-EGFP in red) and wildtype (WT) HEK293 cells (blue) were incubated with 4.5, 15, and 22.5 µg of D7-IgG4. Specifically, bound antibodies were detected with an anti-human IgG4 antibody conjugated with AlexaFluor^®^ 647 (AF647). As negative control, cells were incubated with anti-human IgG4 antibody only (grey). Fluorescence intensities for these cell populations were presented as histograms in AF647 channel

For a further characterization of three binders (15-IgG4, C2-IgG4, D7-IgG4), flow cytometric analyses with the EpCAM cell line were performed. The candidates 15-IgG4 and C2-IgG4 were not able to detect the EpCAM in flow cytometry. For D7-IgG4 a moderate binding of EpCAM cells (44%) in dependence of the concentration could be observed (Fig. 14).

## Discussion

The generation of human recombinant antibodies by phage display is a common technology to create potent biologicals for a variety of therapeutic implications [30–33]. The selection of binders is mainly performed by using ELISA-based screenings where the antigen is coated to a solid phase and binders which sticks to the antigen were eluated in different panning rounds. Depending on the availability and structural composition of the antigen an effective panning and selection of functional binders can be impaired. Especially, membrane proteins with a complicated structural assembly cannot be mimicked entirely by coating the extracellular domain to the solid phase of an ELISA plate. Therefore, different approaches were made using cell lines expressing these complicated targets on the cell surface to provide a native composition for the selection of functional binders [9, 34–38]. Previously, Jones *et al*. published an approach where whole cells expressing the target protein were used in phage display. The expression of the target membrane proteins was linked to intracellular GFP expression which was monitored by flow cytometry and adjusted as internal control for target expression. The authors described that the GFP signal was decreasing after 2 days in culture which correlates to a decreased target protein expression. To enable a stable protein expression over time we made two further adjustments: (i) we used an EFα1 promotor which enables a long-term expression of the target protein and (ii) we used the piggybac transposase system for a stable integration into the genome as described previously [39, 40]. To streamline the detection of bound phages during flow cytometry we decided to use a specific anti-phage antibody labeled with a fluorescent dye. This allowed a high-throughput selection and sorting via flow cytometry instead of pelleting bound phages by centrifugation and elution through a pH shift. To apply this optimized workflow on relevant targets, in this study, human membrane proteins EpCAM and NP65 were chosen and used for the screening of potential binders from an in-house naïve human scFv phage library. In general, it is uncommon to use a phage library of human origin to identify binders against human antigens. However, due to their potential use for therapy or *in vivo* diagnostic it is important to have binders originated from humans. The rationale behind this approach was the development and optimization of a high throughput flow cytometry selection to identify rare binders within a phage pool of human donors to select fully human antibodies. For both antigens it was possible to enrich specific phage populations in flow cytometry and to identify their sequences. The recombinant expressed EpCAM-specific binders with an IgG4 scaffold showed a 50-fold lower binding intensity in an ELISA with the recombinant antigen compared to the control antibody HEA-125. HEA-125 was generated by conventional hybridoma technology with an immunization of the physical antigen over several weeks in mice. Naïve human phage display libraries contain the natural repertoire of sequences given by the donor. Given the nature of the host immune response, the immune system is trained to not react against self-antigens. This mechanism is not a 100% protection as seen in autoimmune conditions where the host is developing antibodies attacking self-structures. The phage display screening we have established here represents an artificial setup which allows the selection of human binders against human antigens. *In vivo* the affinity maturation is taking place by somatic hypermutation and clonal selection. *In vitro*, these processes are refined by different mutation and re-selection approaches to establish sub-libraries and select binders with higher affinities. A common procedure is mutagenesis of antibody sequences by error-prone PCRs [41]. Using a DNA shuffling approach, CDR regions of the antibodies are mutated to create binders with higher affinities. Another approach is an oligonucleotide-directed mutagenesis in which conserved regions of the framework remain untouched but the variable regions are mutagenized to identify candidates with higher affinities [42, 43]. Both approaches can be utilized to the antibody candidates identified in this study to improve their affinities.

The described approach to select phage antibodies targeting complex membrane bound antigens using transgenic cell lines as target and a high-throughput flow cytometric sorting offers an improved outcome of selection rounds and decreases laborious ELISA panning procedures. Furthermore, it reduces the need of recombinantly expressed proteins or extracellular parts of the protein, which influences structure and folding, and, therefore, the selection of binders targeting the native *in vivo* state of the protein on host cells.

## Data Availability

Raw data are available from the authors after request.
